# Dr. Alpheus Prince

**Published:** 1911-07

**Authors:** 


Dr. Alpheus Prince, of Bryon, N. Y., a graduate of the University of Buffalo (1883) died at the home of his son in Ithaca, N. Y., May 18, 1911, of nephritis, aged 55 years. He was a member of the Byron board of pension examining surgeons.

